# Nasoalveolar Molding and Feeding in Infants with Cleft Lip and Palate: An Exploratory Study of Parents’ and Caregivers’ Perceived Experiences

**DOI:** 10.3390/medsci14050573

**Published:** 2026-09-16

**Authors:** Enrique España-Guerrero, Esther Liceras-Liceras, Alba España-Guerrero, Adoración Martínez-Plaza, Ingrid Garzón, Ricardo Fernández-Valadés, Fernando Campos, Antonio España-López

**Affiliations:** 1 Department of Stomatology, Faculty of Dentistry, University of Granada, 18071 Granada, Spain; enrique@dentalos.es; 2 Division of Pediatric Surgery, University Hospital Virgen de las Nieves, 18014 Granada, Spain; esther.liceras.sspa@juntadeandalucia.es (E.L.-L.); rfdezvalades@me.com (R.F.-V.); 3 Doctoral Program in Clinical Medicine and Public Health, University of Granada, 18071 Granada, Spain; alba@dentalos.es; 4 Craniofacial Malformations and Cleft Lip and Palate Management Unit (Unidad de Fisurados Labiopalatinos y Malformaciones Craneofaciales), University Hospital Virgen de las Nieves, 18014 Granada, Spain; adoracionmartinez@medicalpur.com; 5 Division of Oral y Maxillofacial Surgery, University Hospital Virgen de las Nieves, 18014 Granada, Spain; 6 Tissue Engineering Group, Department of Histology, School of Medicine, University of Granada, 18016 Granada, Spain; igarzon@ugr.es; 7 Instituto de Investigacion Biosanitaria ibs.GRANADA, 18012 Granada, Spain

**Keywords:** nasoalveolar molding, cleft lip and palate, feeding

## Abstract

Background/Objectives: Patients with cleft lip and palate (CLP) typically experience feeding difficulties due to an abnormal communication between the oral and nasal cavities, and novel therapies such as palate tissue engineering and nasoalveolar molding (NAM) are in need to improve the final outcomes. This study aims to evaluate the parents and caregivers perceptions on the potential of NAM to improve feeding in patients with CLP. Methods: 42 infants with unilateral or bilateral CLP treated with NAM at a reference craniofacial unit were included in the study. Parents or caregivers completed a four-item questionnaire at 3 months of age, before cheiloplasty, evaluating the effects of NAM to improve nasal regurgitation, choking, air swallowing, and feeding time using a 4-point Likert scale. A total perceived feeding benefit score (range 4–16) was calculated. Results: Parents and caregivers perceived a positive effect of NAM to improve choking (3.67 ± 0.53), nasal regurgitation (3.62 ± 0.58), and feeding time (3.50 ± 0.67), while air swallowing showed lower scores (3.19 ± 0.55). The total perceived feeding benefit score was 13.98 ± 1.57, indicating a high perceived benefit. No significant differences were found between males and females or between unilateral and bilateral clefts. Conclusions: Parents and caregivers consistently perceived a beneficial effect of NAM on patients’ feeding. Although further research is required to determine the actual effectiveness of NAM, examining the perspectives reported by parents and caregivers could contribute to understand treatment experiences and guide family-centered care.

## 1. Introduction

Cleft lip and palate (CLP) represents one of the most prevalent congenital craniofacial malformations, with a global incidence estimated at approximately 1 case per 700 to 1000 live births worldwide, although its prevalence may vary according to several factors, such as the geographic region [1,2]. In general, orofacial clefts comprise a heterogeneous group of conditions including cleft lip, cleft palate and CLP that are typically associated with substantial healthcare needs extending from infancy to adulthood [3]. CLP appears as a consequence of an incomplete embryonic development, with a fusion defect of the facial processes during early craniofacial development, and it may appear as unilateral CLP or bilateral CLP, affecting both sides of the lip [4]. The clinical presentation of this malformation is highly heterogeneous, ranging from a minimal unilateral cleft affecting only the lip to an extensive unilateral or bilateral involvement of the lip, nose, and both the primary and secondary palate [5].

In addition to an important esthetic deformity typically associated with a marked nasal asymmetry and other anatomical defects, CLP is normally accompanied by significant functional impairments affecting feeding, speech, hearing, dental development and facial expression, resulting in a substantial impact on the quality of life of the affected children and their families, leading to significant functional, emotional, and psychosocial consequences [6,7,8]. One of the main functional problems found in patients with CLP is feeding. Effective infant feeding depends on the generation of negative intraoral pressure and the establishment of an adequate seal around the nipple. However, the abnormal communication between the oral and nasal cavities found in patients with CLP prevents the newborn or infant from generating the intraoral pressure required for effective sucking [9]. Therefore, patients are often unable to generate a tight seal around the nipple during breastfeeding, resulting in insufficient negative intraoral pressure during sucking, often resulting in reduced sucking efficiency and impaired milk transfer, and several studies evaluating sucking performance have demonstrated that infants with more extensive clefts generate significantly lower suction pressures than unaffected infants, while feeding ability is closely related to the size and severity of the cleft defect [10,11,12]. Therefore, patients are at increased risk of regurgitation, choking and air swallowing, often requiring prolonged feeding times, resulting in inadequate weight gain and significant stress for parents and caregivers [13]. These functional consequences of CLP often affect patients quality of life and may significantly affect health-related quality of life, for both patients and families and caregivers [14]. For these reasons, a coordinated multidisciplinary care throughout childhood and adolescence is often required by patients with CLP [3].

Feeding difficulties can have important consequences that extend beyond nutrition. In normal conditions, a high percentage of the newborn period corresponds to feeding, and feeding represents a critical opportunity for parent-infant bonding. Persistent feeding difficulties are associated with increased parental anxiety, reduced caregiving confidence, and concerns regarding infant growth and wellbeing. Consequently, feeding impairment is one of the main concerns reported by parents immediately following the birth of a child with CLP [14]. For these reasons, interventions capable of improving feeding efficiency during the presurgical period are of particular clinical interest.

In this context, one of the treatment alternatives that may improve the presurgical situation of children affected by CLP is nasoalveolar molding (NAM), a presurgical orthopedic technique introduced by Grayson and colleagues in the early 1990s [15]. NAM was designed to reduce the severity of the cleft deformity before the surgical repair of the defect, and its application demonstrated to improve and optimize the esthetic and functional outcomes of patients with CLP, by repositioning nasolabial structures, reducing the gap between the alveolar segments and approximating craniofacial structures, thereby facilitating subsequent surgical repair [11,16]. Normally, NAM uses a personalized intraoral molding appliance (intraoral molding plate) combined with a nasal stent. The intraoral molding plate provides the patient with a physical separation between the oral and nasal cavities that may contribute to improving feeding, whereas the nasal stent is used to guide the growth and reposition the alveolar segments, improve nasal symmetry, reduce cleft width, and lengthen the columella in bilateral clefts. All these modifications facilitate the primary surgical reconstruction of the congenital defect, potentially improving the final esthetic and functional outcomes of the patient [17,18].

It is important to note that the success of NAM therapy is strongly dependent on the active participation and adherence of parents or caregivers, as its use is associated with several challenges, particularly in achieving and maintaining an effective latch during breastfeeding [19]. Implication of parents and caregivers is essential, since NAM requires regular appliance use, taping procedures, maintenance, cleaning, and regular visits to the orthodontist and doctors in charge of this treatment that require considerable demands on parents and caregivers. Previous reports demonstrated that treatment success strongly depends on parental compliance and motivation, and treatment discontinuation is often associated with caregiver burden, difficulties managing the appliance, or feeding-related challenges [20]. Therefore, understanding caregiver perceptions regarding the practical benefits of NAM, including its effect on feeding, is essential for evaluating the real-world value of this intervention.

Although NAM is widely used as a presurgical intervention for patients with CLP, the perceptions of parents and caregivers regarding its impact on patient feeding have not been adequately investigated, and relatively little attention has been given to caregiver-reported feeding outcomes. Given the key role of parents and caregivers in the treatment, understanding their perspectives and experiences is essential for optimizing patient- and family-centered care. The objective of this work is to carry out an exploratory analysis to analyze whether parents and caregivers perceived nasoalveolar molding (NAM) therapy as having a positive effect on feeding in infants with CLP.

## 2. Materials and Methods

42 patients with CLP treated at the Unit of Craniofacial Malformations and Cleft Lip and Palate of the University Hospital Virgen de las Nieves were enrolled in the present exploratory study. Inclusion criteria were: (1) patients with non-syndromic unilateral or bilateral complete CLP treated at our Unit from 2024 to 2026; (2) patients who initiated NAM therapy during the neonatal period, that is, from birth and throughout the early weeks of life, at our Unit; (3) written informed consent to participate in the study signed by parents or legal guardians. Exclusion criteria were: (1) syndromic or incomplete CLP; (2) patients admitted at our Unit after 30 days of age; (3) lack of written consent. Enrollment in the study was consecutive, as parents or caregivers of all patients fulfilling the inclusion criteria were invited to participate in the study. This study was approved by the Granada ethics and research committee CEIm (Comité de Ética de la Investigación Provincial de Granada), protocol code SICEIA-2024-002246 (MOLD24), date of approval 29 October 2024, and SICEIA-2025-002550, date of approval 5 November 2025.

Of the 42 patients included in the study, 26.2% (11 out of 42) of the participants were female and 73.8% (31 out of 42) were male. Regarding cleft type, 61.9% (26 out of 42) of cases were unilateral, whereas 38.1% (16 out of 42) had bilateral CLP. Following the protocols established at the Unit, all patients were initially treated using NAM therapy [16]. After this, patients received surgical repair of the lip defect (cheiloplasty) at approximately 6 months of age, whereas correction of the cleft palate (uranostaphylorrhaphy) was performed when the patient was approximately 15–18 months of age. Since all patients treated in our center currently receive NAM therapy, the study was carried out exclusively on families of children with CLP who underwent NAM. Patients treated without NAM were not included in the study. Parents or caregivers were informed by the surgeon and the orthodontist of the Unit on the benefits and limitations of the NAM procedure, and parents or caregivers provided informed consent for the participation of the patients in this study.

Usually, a prenatal diagnosis is carried out in most patients, allowing parents to attend a pre-birth consultation at the Unit, where they receive information regarding the condition and its management. At the moment of birth, newborns with CLP were provided with a nasogastric tube to facilitate feeding, while maintaining uninterrupted skin-to-skin contact and close interaction with their mothers. Within the first 24–48 h after birth, intraoral and extraoral impressions were taken using putty silicone (Virtual Putty^®^, Ivoclar Vivadent, Schaan, Liechtenstein). These impressions were used to fabricate a NAM palatal plate with a combination of hard orthodontic acrylic resin (Leocryl^®^, Leone, Florence, Italy) with soft resin (Eversoft^®^, Austenal, Chicago, IL, USA) [16]. This palatal plate appliance is inserted in the patient’s palate when the patient is less than 1 week old (mean 5.07 ± 3.92 days), with the objective of separating the oral and nasal cavities, promoting alignment of the segments and facilitating patient’s feeding, coinciding with removal of the nasogastric tube [16,17]. The palatal plate is adjusted weekly to adapt it to the patient’s growth, and a nasal extension -two nasal extensions in cases with bilateral CLP- is added to the plate approximately 2–3 months after birth to guide nasal development. All NAM treatments are performed by a single orthodontist, and all the surgical procedures are carried out by the same surgeon, to minimize interoperator variability.

For each patient, parents or caregivers were asked to complete an exploratory non-validated questionnaire designed ad-hoc for the present work, consisting of 4 items evaluating their perceptions of changes in the patient’s feeding following NAM therapy (Supplementary Table S1). The questionnaire was administered in a standardized manner to both parents or caregivers during the consultation performed when the infant reached 3 months of age, prior to cleft lip surgical repair. Parents or caregivers were instructed to complete the survey jointly, ensuring that a single consolidated response document was generated for each patient. The 4 items were:

1. The use of NAM reduces nasal regurgitation during feeding.
2. The use of NAM reduces choking episodes during feeding.
3. The use of NAM reduces excessive air swallowing during feeding.
4. The use of NAM reduces the total time required for feeding.

Responses to each item were rated using a four-level Likert-like scale (scores 1 to 4), where 1 indicated that “the condition became worse after the treatment”, 2 indicated that “the condition remained unchanged, and was not influenced by the treatment”, 3 indicated that “the condition was partially improved by the treatment” and 4 indicated that “the condition had improved significantly after the treatment”. The scores assigned to the 4 items were summed to calculate a total perceived feeding benefit score of the parents or caregivers with the treatment.

Statistical analysis: A sample size calculation was performed based on a two-sided one-sample normal (Z) test, assuming a significance level of 0.05, a statistical power of 80%, and an expected effect size of 0.44. Based on these assumptions, a minimum sample size of 41 patients was required. To determine the consistency of the questionnaire, we first used Cronbach’s alpha reliability coefficient to determine the internal consistency of the test, and values above 0.70 were considered indicative of reliability of the results. Then, we obtained descriptive statistics for each study variable, not only for the whole group of patients, but also for males and females, and for patients with unilateral CLP and patients with bilateral CLP. Before carrying out comparative analysis, we used the Shapiro-Wilk test to determine if each distribution met the normality assumptions for parametric testing, and variance homoscedasticity was determined using the test of Levene. Given that most variables did not follow a normal distribution, non-parametric statistical comparison tests were selected. Comparisons between males and females and between patients with unilateral CLP versus patients with bilateral CLP, were performed using Mann–Whitney U test. Comparisons between results corresponding to two different items were done with the Wilcoxon signed-rank test for paired samples.

For each comparison, *p* values, T statistics and effect sizes (r) were calculated from the Wilcoxon statistical analysis, and median paired differences were obtained for each comparison. Then, 95% confidence intervals were calculated using the Hodges–Lehmann estimator of the difference between groups. All comparisons were carried out two-tailed. A Bonferroni adjustment was applied to the *p*-values to control for inflation of Type I error due to multiple testing, with statistical significance set at *p* < 0.0031. The statistical analyses were made using Purdue University Real Statistics software (Release 7.2) (https://www.real-statistics.com/).

## 3. Results

**Figure 1:**
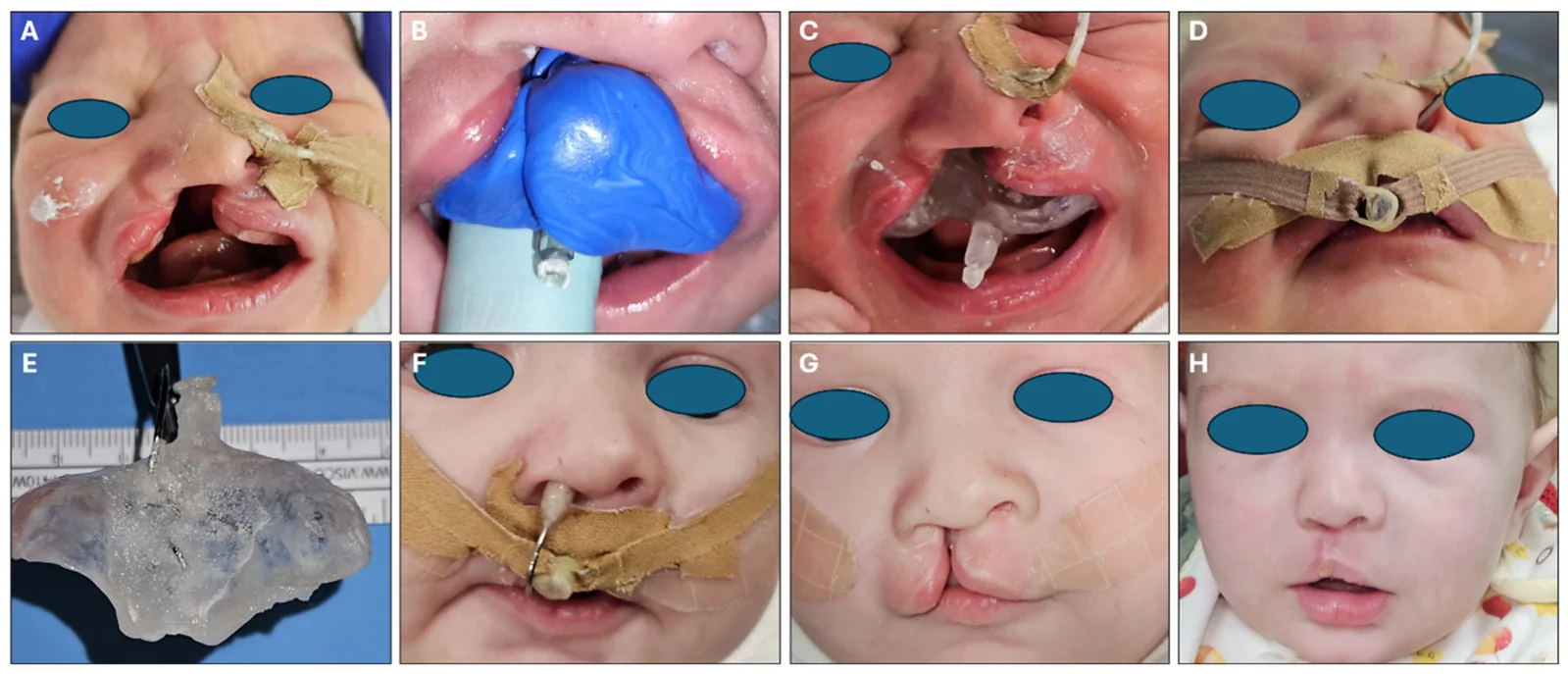
Clinical sequence illustrating the nasoalveolar molding (NAM) treatment process in a male patient with unilateral cleft lip and palate (CLP). (**A**) Initial clinical situation before the treatment, showing the unilateral cleft involving the lip, alveolar ridge, and nasal deformity. (**B**) Obtaining the palatal impressions using silicone material to generate a customized intraoral molding appliance. (**C**) Initial insertion and clinical adaptation of the palatal molding plate. (**D**) Extraoral fixation of the palatal plate using adhesive tapes and orthodontic elastics to ensure adequate retention and continuous orthopedic action of the NAM device. (**E**) Modified palatal plate incorporating a nasal extension designed to support and mold the nasal cartilage and improve nasal symmetry. (**F**) Frontal view of the patient with the palatal plate and the nasal extension in place, demonstrating elevation and support of the nasal dome. (**G**) Clinical appearance immediately before primary cheiloplasty surgery, showing approximation of the alveolar segments, reduction of the cleft gap, and improvement of the nasolabial tissues following completion of the NAM protocol. (**H**) Postoperative clinical results after NAM followed by cheiloplasty, illustrating restoration of lip continuity and improved nasolabial esthetics. The sequential images demonstrate the progressive orthopedic and soft tissue modifications achieved during presurgical NAM therapy and their contribution to facilitating subsequent surgical repair.

**Figure 2:**
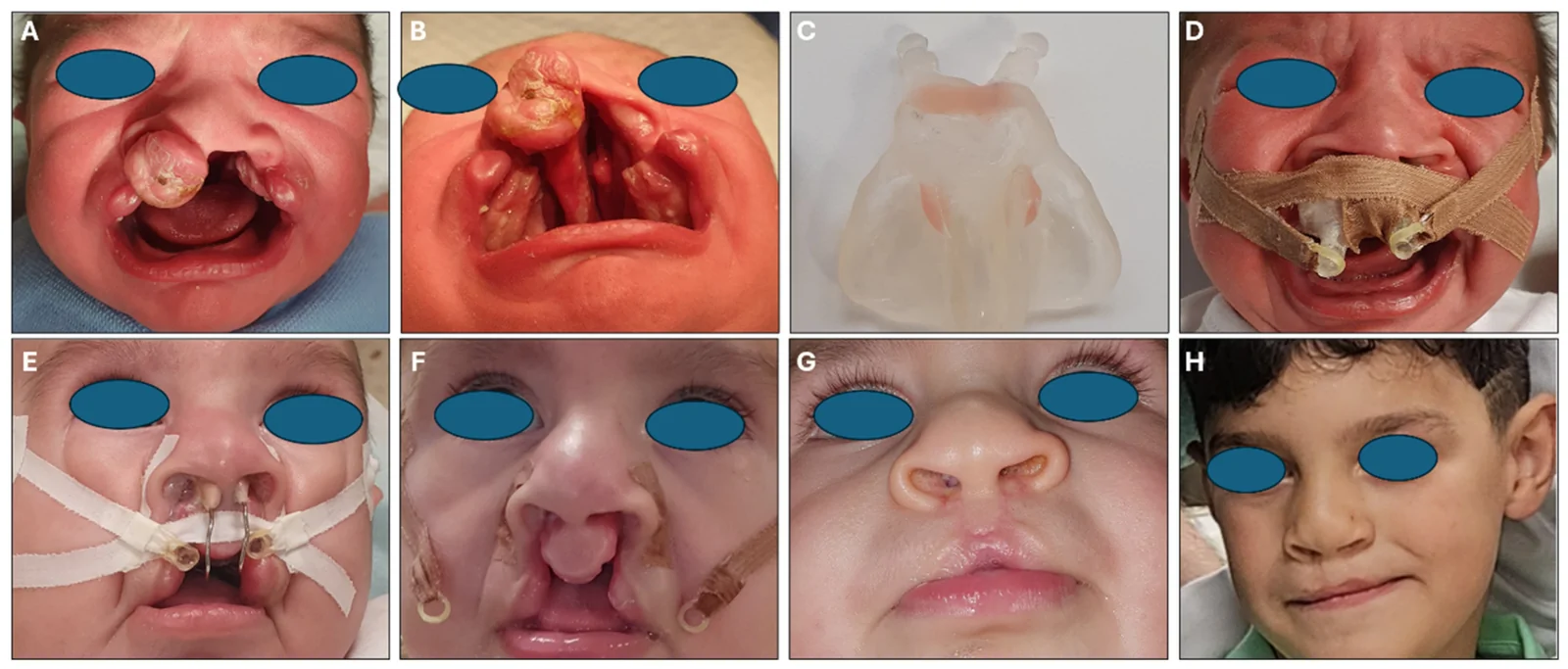
Clinical sequence illustrating the nasoalveolar molding (NAM) treatment process in a male patient with bilateral cleft lip and palate (CLP). (**A**,**B**) Initial clinical situation before the treatment, showing the bilateral cleft involving the lip, alveolar ridge, and nasal deformity, with significant displacement of the premaxilla. (**C**) Palatal plate generated with acrylic resin. (**D**) Extraoral fixation of the palatal plate using adhesive tapes and orthodontic elastics to ensure adequate retention and continuous orthopedic action of the NAM device. (**E**) Modified palatal plate incorporating two nasal extensions designed to support and mold the nasal cartilages and improve nasal symmetry. (**F**) Clinical appearance immediately before primary cheiloplasty surgery, showing approximation of the alveolar segments, reduction of the cleft gap, repositioning of the premaxilla and improvement of the nasolabial tissues following completion of the NAM protocol. (**G**,**H**) Postoperative clinical results after NAM followed by cheiloplasty, illustrating restoration of lip continuity and improved nasolabial esthetics. The sequential images demonstrate the progressive orthopedic and soft tissue modifications achieved during presurgical NAM therapy and their contribution to facilitating subsequent surgical repair.

All patients included in the study completed the NAM therapy without major complications. Overall, all patients with unilateral or bilateral CLP tolerated the treatment very well and acceptance by parents and caregivers was favorable, with good compliance with the treatment. Representative clinical outcome images are shown in Figure 1 and Figure 2, illustrating the reduction of the alveolar cleft gap, improvement in nasal symmetry, and favorable repositioning of the nasolabial tissues after NAM treatment.

When the results of the questionnaire were analyzed to determine reliability of the data, we found that the internal consistency of the results, as determined by Cronbach’s alpha coefficient was 0.7635, indicating that the data were reliable and there was homogeneity among the questionnaire items evaluated in the present work. Overall, these results suggest that the instrument was appropriate for evaluating the perceptions of parents and caregivers regarding the influence of NAM therapy on infant feeding.

Then, our analysis of the results obtained for each item in the questionnaire (Table 1) revealed generally positive responses across all the items, with the highest mean results corresponding to item 2 (3.67 ± 0.53 points), followed by item 1 (3.62 ± 0.58 points) and item 4 (3.50 ± 0.67 points). Statistical comparisons demonstrated that the differences among these three items were not significant (*p* > 0.0031), suggesting a similarly high level of agreement among parents and caregivers regarding these aspects of feeding improvement (Table 2). However, the lowest scores corresponded to item 3, with a mean value of 3.19 ± 0.55 points, with significant differences from item 1 (*p* = 0.0017) and item 2 (*p* = 0.0005), but not with item 4 after Bonferroni correction (*p* = 0.0207), suggesting that the perceived benefit associated with the aspect evaluated by item 3 was less pronounced than that reported for items 1 and 2. When effect sizes were calculated using the rank-biserial correlation, we found moderate effects for item 1 vs. item 3 (r = 0.3934) and item 2 vs. item 3 (r = 0.4333). All other comparisons showed small or very small effects. The total perceived feeding benefit score obtained in the questionnaire was 13.98 ± 1.57 points, suggesting a high overall level of parental satisfaction and a broadly positive perception of the effect of NAM therapy on feeding performance.

To evaluate whether caregiver perceptions differed according to patient characteristics, subgroup analyses were performed based on gender and cleft type (unilateral or bilateral CLP). In this regard, comparison of the questionnaire scores between male and female patients revealed non-significant differences between both genders for all the assessed variables or in the overall satisfaction score (*p* > 0.0031). Similarly, differences among unilateral and bilateral CLP were not statistically significant (*p* > 0.0031). These findings suggest that the perceived feeding benefits associated with NAM therapy were consistent regardless of patient gender or the type of CLP.

## 4. Discussion

Children born with CLP frequently experience feeding difficulties due to impaired coordination of sucking, swallowing, and breathing, which limits their ability to generate adequate negative intraoral pressure [9,12,21,22]. As a result, infants often present prolonged feeding times, excessive air intake, nasal regurgitation, fatigue, and insufficient caloric intake, contributing to poor weight gain and risk of failure to thrive [23]. Feeding difficulties account for 28.4% of requests for surgery [24] and impose substantial physical and emotional strain on parents and caregivers [25]. These feeding challenges highlight the need for early multidisciplinary support involving surgeons, speech and language therapists, lactation consultants, and nutrition specialists [26].

Typically, patients with CLP require specialized feeding interventions, especially during the early postnatal period. These interventions frequently involve the use of different types of feeding appliances, such as palatal obturators or sucking plates, which contribute to creating an effective seal within the buccal cavity and separate the oral cavity from the nasal compartment, thereby facilitating feeding and reducing nasal regurgitation [27]. However, the clinical effectiveness of these devices may be limited, as available evidence suggests variable benefits among patients. Therefore, feeding interventions should not rely exclusively on these appliances, and families should be offered personalized feeding support and specific lactation education programs aimed at optimizing feeding techniques, improving caregiver confidence and supporting adequate nutritional intake [28].

A major shift in cleft palate management is the introduction of advanced therapy medicinal products (ATMP) that offer regenerative alternatives for conditions lacking curative solutions. Among these, bioengineered palate substitutes such as BIOCLEFT represent a promising tissue-engineering approach for patients with CLP. Another promising presurgical intervention for these patients is NAM. By using a palatal prosthesis combined with nasal extensions, NAM significantly contributes to approximate the alveolar segments, and to realign misplaced structures of the patient’s oral cavity, resulting in a more physiological positioning of the maxillofacial structures [29]. NAM has previously demonstrated to significantly improve nasolabial aesthetics, nasal symmetry, columellar length and alveolar alignment in children with CLP [7,16], potentially reducing the complexity of subsequent surgical procedures, resulting in enhanced maxillofacial outcomes [29].

In addition, a positive functional effect of NAM on the sucking ability of patients with cleft palate has been proposed. By contributing to obturate the palatal defect, NAM devices may improve the separation between the nasal and oral cavities, preventing nasal regurgitation and choking, resulting in an adequate nutritional intake before the surgical procedure [9,30,31]. Although these parameters were not evaluated in the present work, this mechanism might contribute to reducing feeding-related fatigue, potentially contributing to a more efficient nutritional intake and improved weight gain before surgical intervention. Despite these potential benefits, the effect of NAM on feeding outcomes remains considerably less studied than its impact on craniofacial morphology and aesthetics, especially regarding the perceptions of the parents and caregivers, who are directly involved in the daily management of feeding difficulties experienced by patients with CLP. Because feeding difficulties directly affect daily care, evaluating families’ perceptions provides valuable insight into the functional impact of NAM, its influence on quality of life, and its role in easing feeding burden during the presurgical period.

In the present work, we have evaluated 42 patients affected by CLP, and we found that the total perceived feeding benefit score of parents and caregivers regarding the ability of NAM to improve feeding reached more than 87% of the maximum possible satisfaction score. Although these findings should be interpreted with caution, given the potential influence of social desirability/expectation bias and survivorship bias, they may suggest that parents and caregivers generally perceived feeding to be easier during NAM therapy. However, this study reflects parents’ and caregivers’ perceptions and subjective experiences rather than objective measurements of feeding-related outcomes, including weight gain, feeding duration, caloric intake, hospital admissions, aspiration episodes and growth percentiles. Therefore, the reported improvements should be interpreted as indicators of parental perceptions, rather than evidence of the clinical effectiveness of NAM in improving feeding outcomes.

Although strong scientific evidence specifically addressing feeding outcomes following NAM remains limited, parents and caregivers perceptions reported in the present work are in agreement with previous reports by Ferreira and cols. [32], who described improved breastfeeding performance after NAM treatment, and with the findings published by Gnaneswar and cols. [33] suggesting that NAM may be associated with superior feeding effectiveness and enhanced weight gain in a preliminary clinical trial. Similarly, Ndem and cols. [34] observed a tendency toward greater growth velocity and body mass index in infants treated with NAM. Although future studies should be carried out to confirm the usefulness of NAM in improving patients’ feeding, we hypothesize that the use of NAM might enhance sucking efficiency and reduce feeding time by creating a more efficient seal around the nipple, thereby facilitating suction, as suggested by previous works using NAM in patients with CLP [35].

In our exploratory subgroup analysis, neither patient sex nor cleft type (unilateral vs. bilateral) showed statistically significant differences in the perceptions reported by parents and caregivers. However, these results must be interpreted with caution, because the small sample size of some subgroups may have substantially limited statistical power. Therefore, the non-significant results are exploratory and should not be interpreted as evidence of equivalent perceptions across sex or cleft type, but should instead be regarded as an inconclusive finding. Although previous works referred gender-related statistical differences between males and females regarding the type of CLP, with males showing higher incidence of CLP and females showing higher percentages of isolated cleft palate [36], most works failed to find significant gender differences regarding the effectiveness of NAM in males and females [16]. Our findings are therefore consistent with previous studies reporting similar treatment effectiveness between male and female patients undergoing presurgical infant orthopedic interventions [16], but given the limited power of our subgroup analyses, they should be considered preliminary. Specifically, the notably higher number of male than female patients included in the study is intriguing, and may be attributable to the relatively small sample size of the present study. Future studies should include larger and more balanced samples to address this imbalance and provide more robust and generalizable findings. Likewise, the absence of differences between unilateral and bilateral CLP patients may suggest that the functional benefits perceived by caregivers are more associated with the restoration of oral competence than with the specific anatomical pattern of the cleft, and that parents and caregivers perceived the same feeding problems in patients with unilateral and bilateral CLP.

Although NAM was primarily designed to assist in the anatomical approximation of alveolar and nasal structures, several authors have proposed additional functional mechanisms that could theoretically influence feeding outcomes in children with CLP. Evidence from palatal prostheses used in other pediatric conditions suggests that continuous intraoral contact can provide sensorimotor stimulation [37], and experimental works highlighted the importance of palatal sensory feedback for sucking and swallowing behaviors [38]. One possible explanation is that the palatal plates of NAM might enhance afferent sensory input to the lips, tongue, and surrounding structures, supporting oral-motor adaptation and potentially contributing to improved tongue positioning, lip seal, and neuromuscular coordination [39]. However, these mechanisms were not assessed in the present study, which relied exclusively on parental and caregiver perceptions. Therefore, the proposed sensorimotor and neuromuscular mechanisms remain theoretical and should be considered hypothetical rather than evidence derived from our data.

The present study has several limitations. First, the study is exploratory and did not include a control group of patients treated without NAM, which could have provided valuable comparative information regarding parental and caregivers perceptions of feeding outcomes using different therapeutic approaches. Future prospective studies should compare the effects of NAM on patient feeding with those of other feeding-support interventions, such as feeding prostheses or palatal plates [40]. Second, the study is based on the analysis of the perceptions and experiences reported by parents and caregivers and did not include an objective analysis of feeding performance. Therefore, results only reflect parents and caregivers’ subjective perceptions. Future research should include objective and quantitative measurements of the impact of NAM on improving feeding duration, caloric intake, weight gain, growth parameters, and aspiration-related events, to better elucidate the relationship between NAM therapy and feeding performance. Furthermore, the present work relies on the results obtained using an exploratory questionnaire that requires further validation. Although this instrument showed acceptable internal consistency, a formal validation process, including assessment of content validity, construct validity and test-retest reliability, is necessary to guarantee the validity of this questionnaire.

In addition, several sources of systematic bias may affect the results of the present study. First, expectation and courtesy (social desirability) bias [41] may have influenced the perceived benefits of NAM reported by participants, as families receive extensive pre-treatment counselling from the surgical and orthodontic team involved in the treatment, which typically emphasizes the potential advantages of the therapy. This information may prime parents and caregivers toward more positive responses. Second, survivorship bias must be considered, since only families who continued NAM until the 3-month consultation completed the questionnaire. Early discontinuation of NAM, which could be associated with feeding difficulties, was therefore not captured, potentially shifting the results toward more favorable perceptions. Finally, the presence of a ceiling effect cannot be ruled out, as the mean scores obtained in this study were close to the upper limit of the 4-point scale, which may reduce sensitivity and may inflate the apparent magnitude of the perceived benefit [42].

Finally, the study was conducted at a single center and involved a relatively small sample size. Additional limitations of the study include the absence of blinded outcome assessment and longitudinal follow-up. Taken together, these limitations highlight the need for larger, multicenter, prospective studies incorporating validated outcome measures, objective feeding assessments, and appropriate comparison groups to better distinguish caregiver perceptions from the true clinical effects of NAM therapy on feeding outcomes.

## 5. Conclusions

In summary, this study suggests that parents and caregivers consistently perceived NAM therapy as having a positive influence on the feeding process of patients with CLP. However, these findings should be interpreted with caution and should not be considered evidence of the effectiveness of NAM in improving feeding outcomes, since the present study was based on the perceptions reported by parents and caregivers, and did not include objective feeding measurements or a control group for comparison. Although future research is needed to determine the actual potential of NAM to improve feeding outcomes, these results highlight the importance of analyzing caregiver perspectives when evaluating treatment experiences and planning family-centered care.

## Figures and Tables

**Table 1:** Descriptive and comparative analysis of questionnaire scores assessing parents and caregivers perceptions regarding the influence of nasoalveolar molding (NAM) therapy on feeding in infants with cleft lip and palate (CLP). Values are expressed as means ± standard deviations. Item 1 corresponds to regurgitation, Item 2 to choking, Item 3 to air swallowing, and Item 4 to feeding time. The total perceived feeding benefit score represents the cumulative score obtained from all the other questionnaire items. Differences between male and female participants, as well as between patients with unilateral and bilateral CLP, were analyzed using the Mann-Whitney U test. Statistical significance was established at *p* < 0.0031. No significant differences were found according to gender or cleft type.

	Item 1: Regurgitation	Item 2: Choking	Item 3: Air Swallowing	Item 4: Feeding Time	Total Perceived Feeding Benefit Score
All patients	3.62 ± 0.58	3.67 ± 0.53	3.19 ± 0.55	3.50 ± 0.67	13.98 ± 1.57
Males	3.61 ± 0.62	3.65 ± 0.55	3.23 ± 0.62	3.58 ± 0.62	14.06 ± 1.61
Females	3.64 ± 0.50	3.73 ± 0.47	3.09 ± 0.30	3.27 ± 0.79	13.73 ± 1.49
Males vs. Females	0.9326	0.7780	0.4979	0.2945	0.3806
Unilateral CLP	3.54 ± 0.65	3.62 ± 0.57	3.15 ± 0.61	3.42 ± 0.70	13.73 ± 1.73
Bilateral CLP	3.75 ± 0.45	3.75 ± 0.45	3.25 ± 0.45	3.63 ± 0.62	14.38 ± 1.20
Unilateral vs. Bilateral CLP	0.5872	0.7112	0.8162	0.5754	0.4635

**Table 2:** Results of the statistical analysis comparing the results obtained for each item evaluated in the present work. *p* and T values were obtained using the Wilcoxon signed-rank test. Confidence intervals (CI) were obtained with the Hodges–Lehmann estimator of the difference between groups. Statistically significant *p* values were highlighted with asterisks (*).

Comparison Groups	Wilcoxon Signed-Rank *p* Value	Wilcoxon T Statistic	Median Paired Difference	Effect Size (r)	95% CI
Item 1 vs. Item 2	0.7057	60.5	0	0.0983	(0.00; 0.00)
Item 1 vs. Item 3	0.0016 *	50.0	1	0.6773	(0.00; 1.00)
Item 1 vs. Item 4	0.3926	90.0	0	0.2084	(0.00; 0.00)
Item 2 vs. Item 3	0.0002 *	23.0	1	0.8143	(0.00; 1.00)
Item 2 vs. Item 4	0.1688	60.0	0	0.3632	(0.00; 0.00)
Item 3 vs. Item 4	0.0190	55.0	0	0.5504	(−1.00; 0.00)

## Data Availability

The data presented in this study are openly available in the European repository Zenodo at https://doi.org/10.5281/zenodo.21426933.

## Supplementary Materials

The following supporting information can be downloaded at: https://www.mdpi.com/article/10.3390/medsci14050573/s1, Table S1: Questionnaire used in the present work. Parents or caregivers were asked to rate their perceptions regarding the effects of nasoalveolar molding (NAM) during infant feeding across 4 items by placing an “X” in the corresponding response box.

## Abbreviations

The following abbreviations are used in this manuscript:

CLP	Cleft lip and palate
NAM	Nasoalveolar molding
