# Supplementary material for: Nasoalveolar Molding and Feeding in Infants with Cleft Lip and Palate: An Exploratory Study of Parents’ and Caregivers’ Perceived Experiences
Source: Med Sci (Basel). 2026 Sep 16;14(5):573. doi: 10.3390/medsci14050573 (PMC13609556; doi:10.3390/medsci14050573)
Supplement: Supplementary file 1 [file medsci-14-00573-s001.zip › medsci-4539995-supplementary.pdf]

Supplementary Table S1. Questionnaire used in the present work. Parents or caregivers were asked to rate their perceptions regarding the effects of nasoalveolar molding (NAM) during infant feeding across 4 items by placing an “X” in the corresponding response box.

Item	Strongly disagree (1)	Disagree (2)	Neutral (3)	Agree (4)	Strongly agree (5)
**1. The use of NAM reduces nasal regurgitation during feeding.**					
**2. The use of NAM reduces choking episodes during feeding.**					
**3. The use of NAM reduces excessive air swallowing during feeding.**					
**4. The use of NAM reduces the total time required for feeding.**					
